# Evaluating the effects of smoking on the voice and subjective voice problems using a meta-analysis approach

**DOI:** 10.1038/s41598-020-61565-3

**Published:** 2020-03-13

**Authors:** Haewon Byeon, Seulki Cha

**Affiliations:** 1 Department of Speech Language Pathology, College of Health Science, Honam University, Gwangju, Republic of Korea; 2 Department of Rehabilitation, Graduate School, Honam University, Gwangju, Republic of Korea; 3 Present Address: Department of Speech Language Pathology, School of Public Health, Honam University, 417, Eodeung-daero, Gwangsan-gu, Gwangju, Republic of Korea

**Keywords:** Lifestyle modification, Health policy, Patient education, Risk factors

## Abstract

The objectives of this study were to identify the effects of smoking on the voice of smokers and present the baseline data for establishing the basis for preventing voice disorders. This study was evaluated using a meta-analysis from studies published between Jan 1, 2000, and Nov 15, 2018. As a result, the final meta-analysis was conducted using nine papers. The standard mean difference was analyzed after dividing the effects of smoking on voice into the pitch (F0), sound quality (jitter, shimmer, and noise to harmonic ratio; NHR), Maximum Phonation Time (MPT), and subjective voice problem. The results showed that there was a significant difference in F0 and MPT. On the other hand, the jitter, shimmer, NHR, and Voice Handicap Index (VHI) had different mean effect size but they were not significantly different. The analysis by sub-function of VHI results showed that the mean effect size was significantly different only in VHI-P (Physical). This study evaluated the effects of smoking on voice using meta-analysis. It was confirmed that smoking had significant and moderate effects on the F0 of voice, MPT, VHI, and physical functions. It is necessary for future meta-analysis studies to conduct randomized controlled experiments or longitudinal studies to confirm the effect sizes of variables.

## Introduction

The physiology of healthy voice production it is affected by a range of factors such as the strength, quality, soundness, and fluidity of sound^1^. However, the changes in the anatomical structure of the larynx can cause functional problems and, as a result, it can negatively affect the voice production to result in voice disorders. It is widely known that voice disorder has a very high reoccurrence rate. A *Lyberg-Åhlander et al*.^2^ evaluated the voice disorders in Sweden and reported that 16.9% of adults (≥18 years old) experienced a voice disorder. Moreover, it was found that 21.9% of adults in the US also experienced a voice disorder in their lifetime^3^. Also, 73.3% of them suffered from the voice disorder multiple times^3^. Particularly, even if voice rehabilitation (e.g., vocal hygiene) is performed after the occurrence of a voice disorder, the risk of a voice disorder reoccurrence is even higher unless the risk factor negatively affecting voice is removed. Although many studies have identified a relationship between chronic smoking and laryngeal pathology, there is still insufficient evidence of the effects of smoking on voice.

Previous studies on the effects of smoking on voice health can be divided into three major groups. First, smoking has been reported as a representative risk factor of voice health^4^. For example, smoking can cause a g laryngeal disease^5^. Previous studies evaluating the effects of smoking on the larynx using rats and pigs showed that smoking directly caused the anatomical changes of the larynx^6,7^. Moreover, smokers had a higher risk to have a laryngeal disease than non-smokers^4,5^. It was also confirmed that persisted smoking could cause a vocal cord disease such as laryngitis, Rheinke’s edema, and leukoplakia^1^. It was also found that smokers have a risk of various cancers such as oral cancer, pharyngeal cancer, and laryngeal cancer than non-smokers^1,4^.

Secondly, smoking is known to change voice characteristics^8–11^. Smoking induces acoustical changes such as fundamental frequency, jitter, shimmer, and NHR, which determines the quality of voice^12–14^. Moreover, smokers experience more voice use fatigue and more frequent discontinuation of voice use than non-smokers^15,16^. It was found that smokers had a higher abnormality rate in the symmetry, amplitude, and cycle of vocal cord than non-smokers^17^. A recent study also reported that electronic cigarette significantly affected the sound quality such as shimmer and harmonic to noise ratio (HNR)^11,18^.

Third, smoking affects the subjective perception of voice problems. *Pinar et al*.^17^ reported that smokers had higher voice use fatigue and more frequent voice interruption than non-smokers. They also showed that severer voice problems in the self-report type voice evaluation^14^. In summary, smoking may negatively affect subjective health perception as well as objective voice (e.g., sound quality)^9,10^.

Although various studies have been conducted to evaluate the relationship between voice and smoking clearly, there still is not enough evidence to establish the relationship between smoking and voice^5,19^. Even though most previous studies comparing the voice problems of smokers and non-smokers showed that smoking significantly and negatively influenced voice^11,12,17,20,21^, epidemiological studies revealed that smoking was not an independent factor affecting the occurrence of a voice disorder^19^. Therefore, it is needed to examine the relationship between smoking and voice by integrating the results of diverse scientific studies. The objective of this study was to identify the effects of smoking on the voice of smokers and to establish the basis for preventing voice disorders.

## Methods

### Literature review and keywords

Related literatures were collected using four international database (i.e., Pubmed, Ebsco (Academic Search Premier), CINAHL, Science direct) and five Korean database for Korean studies (i.e., DBpia, Korean studies Information Service System, E-article, SCHOLAR, KOREA SCHOLAR) in order to evaluate the effects of smoking on voice. The search was limited to studies published between Jan 1, 2000, and Nov 15, 2018. The search keywords are as follows (i.e., Dysphonia, Dysphonation, Phonation disorder, Voice disorder, Voice less, Voice handicap, Smoking cessation, Smoking water pipes, Tobacco smoking, Pipe smoking, Cigarette smoking, Cigar smoking).

### Including and excluding criterions

This study excluded studies which did not use a statistical analysis such as case studies and reviews. Secondly, only studies with a control group were included. Thirdly, studies dealt with respiratory diseases or neurological diseases (e.g., vocal cord paralysis due to stroke). Fourthly, intervention studies to evaluate the effects of treatment and animal experiments were excluded. This study only examined papers published in English and Korean. The exposed group was defined as the current smokers, while the unexposed group (control group) was defined as people who never smoked.

**Figure 1:**
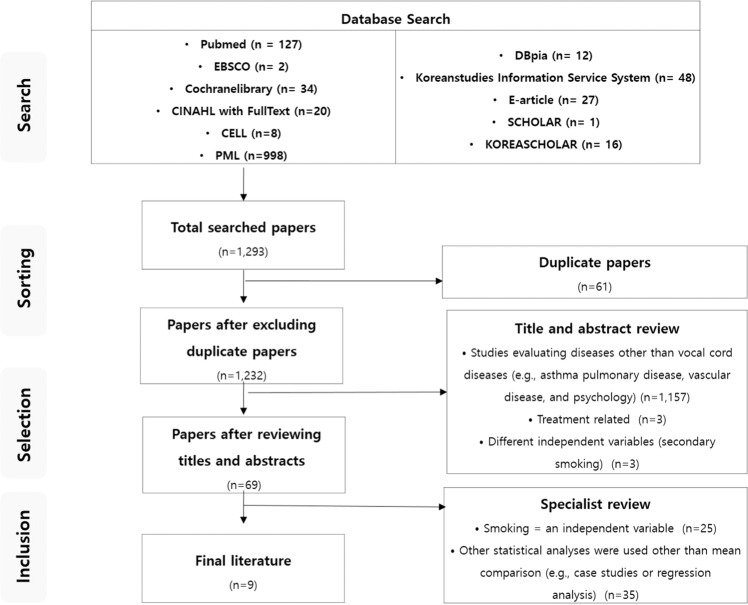
Flow Chart.

A total of 1,293 papers were initially selected for the meta-analysis. Afterward, duplicated papers (n = 61), studies meeting the exclusion criteria such as cases studies (n = 1,163), and studies using median instead of mean and those which we could not examine the full contents (n = 60) were excluded from the study. As a result, the final meta-analysis was conducted using nine papers (Fig. 1).

### Qualitative assessment

The quality of selected literature was assessed using the Newcastle-Ottawa Quality Assessment Scale (NOS). The NOS, developed by the University of Newcastle and University of Ottawa, is a tool to evaluate the quality of control cohort studies^22^. It is composed of four items for evaluating selection and comparability possibility and three items for examining results. The items for selection and result can have one star (★) and the items of comparability can have up to two stars. The more stars means the higher quality of a study (Table 1). The target publications used for the analyses were categorized according to authors, year of publication, number of study subjects, and statistical values (i.e., mean and standard deviation) and coded accordingly. The quality of the literature was assessed by two researchers independently through NOS. When there was disagreement, it was concluded through discussion. The quality evaluation of the target studies ranged from four to seven and only one publication^15^ was excluded from the study due to low quality (Table 2).

**Table 1:** Newcastle-Ottawa Quality Assessment Scale.

Selection
(1) Is the case definition adequate?
(a) yes, with independent validation: reference to IDF (2006) or WHO (1998) or EGIR (1999) or NCEP–ATPIII (2001) or AHA (2004) ★:
If the variable is continuous variable, then it is possible to be omitted.
(b) yes, eg record linkage or based on self reports
(c) no description
(2) Representativeness of the cases
(a) consecutive or obviously representative series of cases: all cases in a defined catchment area, or an appropriate sample of those cases (e.g. random sample) ★
(b) potential for selection biases or not stated
(3) Selection of Controls
(a) community controls ★
(b) hospital controls
(c) no description
(4) Definition of Controls
(a) no history of metabolic syndrome ★
(b) no description of source
**Comparability**
(1) Comparability of case and controls on the basis of the design or analysis
(a) study controls for age and sex (select the most important factor) ★:
It is considered to be adjusted if the scope of adult age is within five.
(b) study controls for any additional factor (SES or educational level or BMI or
weight or height or WC) ★
**Exposure**
(1)Ascertainment of exposure
(a) secure record (eg clinical records) ★
(b) structured interview where blind to case/control status ★
(c) interview not blinded to case/control status
(d) written self report
(e) no description
(2) Same method of ascertainment for cases and controls
(a) yes ★
(b) no
(3) Non-Response rate
(a) same rate for both groups ★
(b) non respondents described
(c) rate different and no designation

**Table 2:** Results of Newcastle-Ottawa Quality Assessment Scale.

Author (Year)	Selection				Comparability	Ecposure			Score
	case definition adequate?	Representativeness of the cases	Selection of Controls	Definition of Controls		Ascertainment of exposure	Same method of ascertainment for cases and controls	Non-Response rate	
Tafiadis *et al*. (2017)	★			★	★	★	★	★	6
Simberg *et al*. (2015)	★				★		★	★	4
Pinar *et al*. (2016)	★			★	★ ★	★	★	★	6
Glas *et al*. (2008)	★			★	★ ★	★	★	★	7
Hamdan *et al*. (2011)	★			★	★ ★	★	★	★	7
Awan. (2011)	★			★	★	★	★	★	6
Hamdan *et al*. (2010)	★			★	★ ★	★	★	★	7
Lee *et al*. (2008)	★			★	★	★	★	★	6
Gonzalez *et al*. (2004)	★			★	★	★	★	★	6

### Quality assessment of target papers

According to the quality evaluation of each item showed that all studies did not secure the representativeness of the exposure groups and the comparison groups (Table 2). Most studies recruited study subjects from hospitals^12^ or a specific institute such as a school^13,15,17,20,21,23,24^ (88.9%). *Hamdan et al*.^25^ did not mention study subject recruitment (11.1%). Five studies (55.6%) defined non-smokers as people who stopped smoking 1year or more. *Simberg et al*.^15^ did not provide the definitions of smokers and non-smokers but the study was conducted by distinguishing between smokers and non-smokers. The only study^24^ only recruited smokers and it did not define non-smokers. Among the nine studies, one study did not report a laryngeal disease. Seven studies (77.8%) evaluated a laryngeal disease and *Hamdan et al*.^24^ directly confirmed the presence of a laryngeal disease using laryngeal stroboscopy.

Five studies have controlled sex^13,15,21,24,25^, while *Awan*^23^ and *Tafiadis et al*.^20^ only targeted female subjects and *Pinar et al*.^17^ and *Lee et al*.^12^ only examined male subjects. *Gonzalez et al*.^13^ evaluated both male and female subjects. Three studies (33.3%; *Hamdan et al*.^24^, *Pinar et al*.^17^, and *Gonzalez et al*.^13^) controlled age.

As a scale to evaluate voice problems in a self-reporting pattern, three studies (37.5%) used the voice handicap index (VHI)^17,20,21^. *Simberg et al*.^15^ (12.5%) only reported the prevalence of a voice disease using a questionnaire that evaluates the presence of a voice disease based on symptoms.

Acoustic evaluation tools were conducted in six studies (66.7%). Among them, two studies (22.2%) used the Visi Pitch system^24,25^, the one study (11.1%) used the Praat software package^17^, one study (11.1%) used Dr. speech software^20^, one study (11.1%) used Multi-Dimensional Voice Program^13^, and *Awan*^23^ (12.5%) used the Dysphonia Severity Index (DSI). All studies used the same interventions to compare groups, and no dropout rate was reported.

### Meta-analysis

The effect size was analyzed using ‘meta’ package of R (ver. 3.4.4). The means and standard deviations of the exposed group and the unexposed group were used in the analysis. For the effect size, standard mean difference (SMD) was used by using Hedge’s g. The weighted mean effect size considering the sample size was used for the mean effect size. The effect size and significance was examined based on the 95% confidence interval. The effect size was divided into ‘small effect (≤0.2), ‘intermediate effect (>0.2 and <0.8)’, and ‘large effect (≥0.8)’.

Homogeneity test was conducted to examine the statistical heterogeneity of the effect size of each study. The results showed that Q-df was higher than 0 and F was confirmed to be equal to or greater than 75%, which implied that the variances of groups were significantly different and the effect size of them was heterogeneous. Therefore, the random effect model was used for this meta-analysis.

The publication bias was examined in order to verify the validity of the meta-analysis results. The funnel plot and adjusted funnel plot revealed that studies were scattered near the effect estimates and it was determined that there was no publication bias. The trim-and-fill method was applied for adjusting visual asymmetry, and it was found that the risk ratios before and after adjustment were similar. Egger’s regression test and Kendall’s tau test were performed to analyze the statistical symmetry. The results showed that it was significantly different in the continuous correlation condition (tau=0.03; p < 0.001). In summary, there was a publication bias error in the publications used for this study. The fail-safe N was calculated to evaluate the level of error and it was found that the fail-safe N was 5.0, indicating a marginal publication bias error.

## Results

### Meta-analysis

#### Mean effect size on the effect of smoking on voice

**Figure 2:**
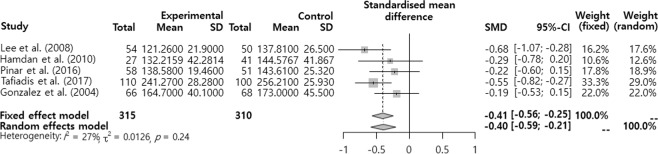
Forest plot for F0.

**Figure 3:**
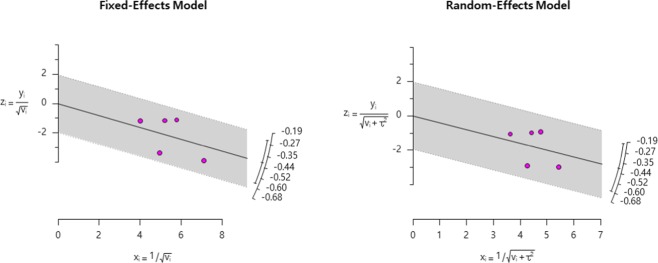
Radial Plot for F0.

**Figure 4:**
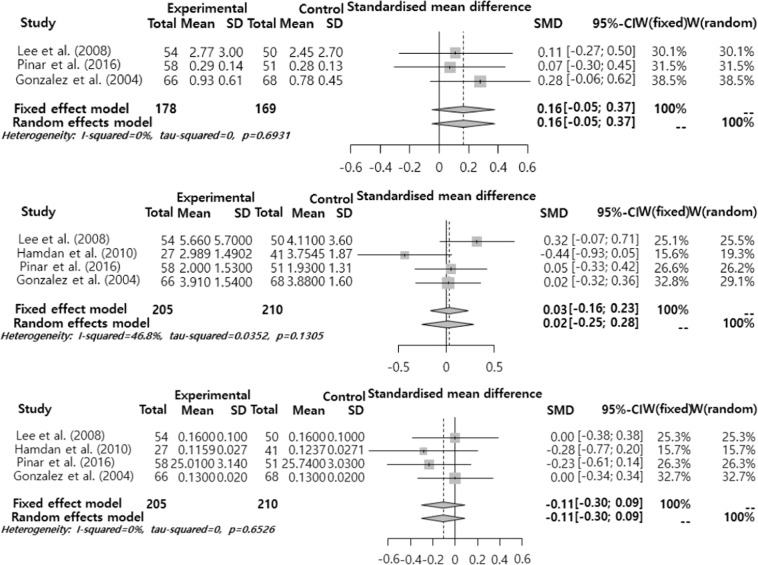
Forest plot on Jitter, Shimmer, NHR (from top to bottom).

**Figure 5:**
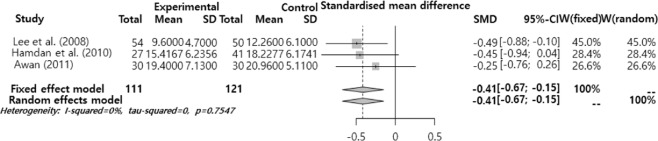
Forest plot for MPT.

**Figure 6:**
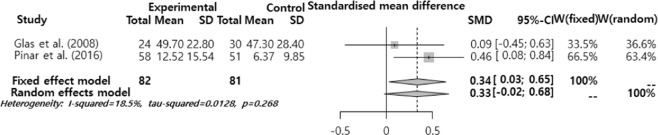
Forest plot for VHI.

The SMD was analyzed after dividing the effects of smoking on voice into the pitch (F0), sound quality (jitter, shimmer, and NHR), MPT, and subjective voice problem. The results showed that there was a significant difference in F0 and MPT (Figs. 2–6). F0 was −0.40 (95% CI = −0.59, −0.21) and there was a significant difference in the ‘intermediate’ level (p < 0.001). Moreover, MPT was −0.41 (95% CI = −0.67, −0.15) and there was a significant difference in the ‘intermediate’ level (p = 0.002). On the other hand, the jitter was 0.16 (95% CI = −0.05, 0.37, p = 0.693), shimmer was 0.02 (95% CI = −0.25, 0.28, p = 0.130), NHR was –0.11 (95% CI = −0.30, 0.09, p = 0.652), and VHI was 0.33 (95% CI = −0.02, 0.68, p = 0.068). They had different mean effect size but they were not significantly different.

#### Subjective voice problems and the effect size of smoking by sub-functions

**Figure 7:**
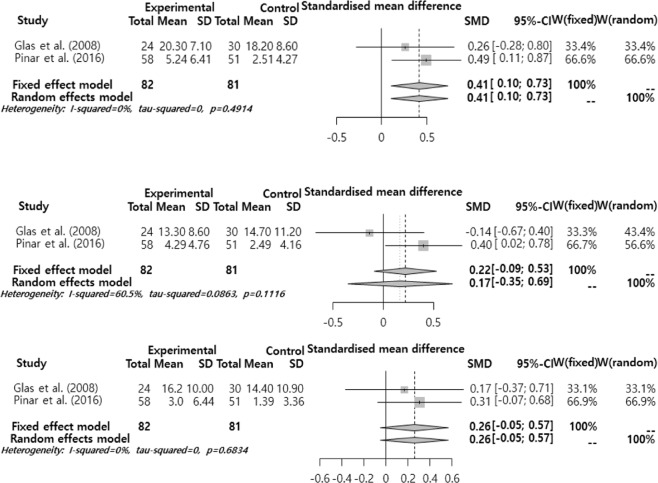
Meta sub-analysis of VHI-Physical, VHI-Functional, VHI-Emotional (from top to bottom).

The effect size of subjective voice problems was analyzed by sub-function (Fig. 7). The analysis results showed that the mean effect size was significantly different only in VHI-P. VHI-P was 0.41 (95% CI = −0.10, 0.73), showing an ‘intermediate’ level significant difference (p = 0.009). On the other hand, VHI-F (Functional) was 0.17 (95% CI = −0.09, 0.53, p = 0.165), and VHI-E (Emotional) was 0.26 (95% CI = −0.05, 0.57, p = 0.099), showing different mean effect sizes but not significant different.

## Discussion

Many previous studies have shown that smoking is a causal factor changing voice and increasing the prevalence of voice disorders^4,5,9,10^. However, there are studies indicating that smoking does not affect the voice^19^. There was heterogeneity in the results of these studies. Therefore, this study conducted a systematic literature review and meta-analysis in order to identify scientific basis regarding the harmful effects of smoking on voice health. This study analyzed the experimental studies published between 2000 and 2018 evaluating the effects of smoking on voice using control groups.

The results of quality evaluation analysis ranged between 4 and 7 points, showing that there was no critical issue to exclude publications except one study. The study with the lowest score evaluated the voice symptoms between smokers and non-smokers using questionnaires, and it did not satisfy the subject selection criteria, comparison possibility, and results criteria^15^. However, it was not excluded from this study because it showed the voice symptoms according to gender and smoking.

It was found that Dr. Speech, Visi-pitch, DSI, Praat, and CSL were the tools used to measure the acoustic characteristics. VHI was used to evaluate the subjective voice. Laryngeal stroboscopy was used to check the symptoms in the larynx. Among them, VHI is a frequently used tool for evaluating voice.

The effect sizes of the nine publications included in this study were analyzed and it was found that smoking only affected the pitch and MPT of voice. Smoking may cause voice disorders by affecting voice directly^23,26,27^. It is related to the organic voice disorders such as laryngeal cancer, rather than functional voice disorders due to the abuse and misuse of voice^4,26,28^. Excessive smoking can irritate the vocal cords and dry the vocal cord mucosa. It can results in inflammation on the vocal cords. It can cause coughing, sputum, and vocal cord feeling of irritation. It may lead to changes in voice^29^. *Pinar et al*.^17^ argued that smoking would increase the weight of the vocal cords, which in turn decrease the fundamental frequency. Moreover, smoking weakens pulmonary function, which can reduce the mean MPT time. *Awan*^23^ examined the relationship between DSI and smoking using 30 women aged between 18 and 24 years, and reported that smoking was strongly related to fundamental frequency decrease and shortened MPT. It is difficult to exclude the possibility that the difference in the algorithms of the evaluations tools could affect the results of this study, because the nine studies included in this study used different acoustic evaluation tools such as CSL, Dr. Speech, Visi pitch, and Praat. It means that future meta-studies should include more studies because the interpretation of the results from these nine studies could be limited.

The effect size was compared by dividing VHI into sub-indices, and the results showed that there were significant differences only in physical functions. VHI is a self-reporting assessment tool that evaluates the voice subjectively. Therefore, it has the potential to underestimate or overestimate the voice problem^25,30^. Also, perceptions about the impact of smoking on their voice varies^14^. Nevertheless, Tafiadis *et al*.^14^ reported that acoustic parameters were correlated with VHI scores, which was similar with the results of this study. Particularly, they showed that NHR and jitter variables were significantly correlated with VHI function and VHI emotional.

The importance of this study was that this study established a scientific foundation to identify the relationship between voice and smoking by summarizing the results of studies evaluating the effects of smoking on voice. The limitations of this study are as follows. First, it is possible that this study did not include studies published in languages other than English and Korean (e.g., Spanish and German), although this study included diverse studies through three international research databases and five domestic research databases. Second, the generalization of the results should be careful because there were less than ten studies in this meta-analysis. Future studies need to conduct more in-depth analyses including more studies. Third, the tools for speech analysis of the studies included in this meta-study were inconsistent. Therefore, there are limitations in interpreting the results. Fourth, it is difficult to track the long-term effects of smoking on voice because all eight studies included in this study were cross-sectional studies. Longitudinal studies are needed to identify the relationship between smoking and voice.

## Conclusion

This study evaluated the effects of smoking on voice using meta-analysis. It was confirmed that smoking had significant and moderate effects on the fundamental frequency, MPT, VHI, and physical functions. However, the studies included in the meta-analysis randomly sampled subjects from a specific institute, so the representativeness of the sample still have limitations. It is necessary for future meta-analysis studies to conduct randomized controlled experiments or longitudinal studies to confirm the effect sizes of variables.
